# Treatment of hypertrophic scars and keloids using an intralesional 1470 nm bare-fibre diode laser: a novel efficient minimally-invasive technique

**DOI:** 10.1038/s41598-020-78738-9

**Published:** 2020-12-10

**Authors:** Ke Li, Fabio Nicoli, Chunxiao Cui, Wen Jing Xi, Ahmed Al-Mousawi, Zheng Zhang, Alberto Balzani, Lindsay Neill, Roberto Sorge, Yun Tong, Yixin Zhang

**Affiliations:** 1 Division of Reconstructive Microsurgery, Department of Plastic and Reconstructive Surgery, Shanghai Ninth People’s Hospital, Shanghai JiaoTong University School of Medicine (China), 639 Zhi Zao Ju Road, Shanghai, 200011 China; 2 Department of Plastic and Reconstructive Surgery, University of Rome “Tor Vergata”, Rome, Italy; 3 Department of Plastic and Reconstructive Surgery, Royal Victoria Infirmary Hospital NHS Foundation Trust, Newcastle upon Tyne, UK; 4 Translational and Clinical Research Institute, Newcastle University, Newcastle upon Tyne, UK; 5 Department of Facial Plastic and Reconstructive Surgery, ENT Institute, Eye and ENT Hospital, Fudan University, Shanghai, China; 6 NHC Key Laboratory of Hearing Medicine, Fudan University, Shanghai, China; 7 Northumbria University, Newcastle upon Tyne, UK; 8 Laboratory of Biometry, University of Rome “Tor Vergata”, Rome, Italy; 9 Department of Medical Cosmetology Surgery, Jinhua People’s Hospital, Jinhua, China

**Keywords:** Biotechnology, Diseases, Health care, Medical research, Pathogenesis, Signs and symptoms

## Abstract

Hypertrophic and keloid scars result from abnormal wound healing and can have a variable response to a number of available treatment modalities. The evolution of laser treatments in recent years has shown a wide range of clinical applications including their use in the treatment of scars. We investigated the effectiveness of a 1470 nm diode laser using an intralesional optical fibre delivery device in the treatment of hypertrophic and keloid scars. We evaluated its safety and efficacy as a novel and minimally invasive treatment alternative for scar modulation and volume reduction. A prospective cohort study was performed involving 21 patients with hypertrophic scars (HS) (n = 9) and keloids (n = 12) resulting from various aetiology. Patients were treated with one to three treatment sessions. Comprehensive evaluations were performed using the Vancouver Scar Scale, Doppler ultrasound, Cutometer, Mexameter and PeriCam PSI. Scar thickness was reduced by an average of 0.308 ± 0.138 cm (*p* < 0.001). In particular the two subgroups showed a significant 27.7% and 28.2% reduction in scar thickness of HS and Keloids, respectively. Scar firmness showed a significant improvement of 1.2% (*p* < 0.05) for HS, though for keloids this was 0.4% (*p* = 0.26). Keloids had a significant reduction in pigmentation at 21.3%. Blood perfusion had a significant reduction of 29.6% in HS and 22.7% in Keloids. Overall VSS total score improvement of 42% in the HS and at 37.9% in the Keloid subgroup. No adverse events such as hypo/hyperpigmentation, skin infection, or recurrence were reported. This study shows that the intralesional 1470 nm bare-fibre diode laser significantly improved hypertrophic and keloid scars based on both subjective and objective analyses and supports this type of laser therapy as a safe and effective minimally-invasive treatment option.

## Introduction

Hypertrophic Scars (HS) and Keloids (K) are the result of abnormal wound healing and scar formation. These pathological scars present as a continuous inflammation and histologically are characterized by fibroblasts proliferation, newly formed blood vessels and collagen deposition. HSK are distinguished clinically by the extent of tissue overgrowth with respect to the margins of the original wound. HS typically occur within a few months in areas were scars are under tension, including the sternal region, around joints and can persist along the margins of the original wound. Keloid scars occur more frequently in individuals with darker pigmented skin, can develop up to several years following minor trauma, and as well develop in areas such as earlobes, shoulders and chest. Keloids are more often symptomatic, being associated with pain and itching, and can proliferate well beyond the original limits of the wound^1,2^. HSK can occur following different injury mechanisms including skin injuries, burns, surgery, injections and dermatitis. Among them, deep burns are reported to be the main cause of HSK^3,4^.

Various treatments have been implicated in their management, however there is still no completely satisfactory technique for treating these scars. A number of different therapies have been proposed such as silicone sheet and gel application, pressure garments, topical and intralesional injections of agents including corticosteroids, interferon, bleomycin, five-fluorouracil (5-FU), as well as cryotherapy, laser, radiotherapy and surgery^5–7^. Despite the wide range of treatments, the reported recurrence rates for keloids remain high at between 50 to 80% and around 10% for hypertrophic scars^8,9^.

In the first instance non-surgical treatment options are preferred. However, if failed, surgery, laser and radiation therapies are considered favourable treatment options^10^.

Although minimally-invasive methods have demonstrated good outcomes for soft and thin scars, there are difficulties for harder textured and thicker scars. Topical medications scarcely penetrate, and injection of therapeutic agents are obstructed by fibrotic tissue, requiring multiple treatments with often limited efficiency^11^. Traditional laser diffusion has also limited efficacy and penetrance within thicker scars. HSK showed positive response to chemotherapeutic agents but adverse effects include pain, ulceration, burning and hyperpigmentation^12^. Surgical excision for HSK reduction remains an option but unfortunately long-term results can be poor, with a high risk of scar recurrence. Currently, no single method provides a complete benefit with recent publications showing contradicting results^5,6,10^. Moreover, the treatment of HSK remains a clinical challenge.

This study is based on our previous positive experience using the 1470 nm diode laser and fibre system to treat inflamed and infected keloids^13^. We demonstrated that the 1470 nm diode laser was able to deliver through the fibre system a localised heating effect within a narrow range. The fibrotic scar tissue rapidly vaporises, instigating cell lysis, necrosis and solidification, that results in tissue ablation and inflammation control. Furthermore, the 1470 nm laser was able to coagulate blood vessels, reducing blood supply and local tissue vascularization^14–16^.

The aim of this research study was to evaluate the efficacy and safety of the 1470 nm fibre laser as a novel method for scar volume reduction. Its effectiveness was assessed in the treatment and improvement of HSK.

## Methods

### Patients

A prospective, single-center cohort study was performed to evaluate the efficacy of a fractional non-ablative diode laser and fibre to treat patients with HSK in various anatomical regions caused by trauma, burns, acne or surgery. Twenty-one patients were observed over a 2-year period between January 2017 and January 2019 treated at the Ninth People's Hospital in Shanghai. All cases were previously treated for more than 1 year with different therapies such as steroid ointments or silicone tapes, but improvements were not observed. Inclusion criteria for the study were a hypertrophic scar or keloid recalcitrant to other therapies with the size of scar more than 10 cm^2^; caused by burns, trauma, surgery, insect bite or acne.

Exclusion criteria were as follows: patients who had undergone previous scar therapies such as laser or surgery, had heart or kidney failure, received immunosuppressive drugs or had autoimmune disorders or uncontrolled diabetes, and those unable to follow the protocol.

### Scar evaluation

Subjective assessment of the scar was evaluated by an experienced independent evaluator pre-operatively and at 6 and 12 months after treatment using the Vancouver Scar Scale (VSS) score. The VSS grading score includes pigmentation (0, normal; 1, hypopigmented; 2, mixed pigmentation; 3, hyperpigmented), vascularity (0, normal; 1, pink; 2, red; 3, purple), pliability (0, normal; 1, supple; 2, yielding; 3, firm; 4, banding; 5, contracture), and height (0, flat; 1, ≤ 2 mm; 2, 2–5 mm; 3, ≥ 5 mm). The score for each parameter was calculated separately, and then all 4 parameters were combined^17^. A questionnaire was completed to study the impact of the scar on quality of life (QOL)^18^. Adverse effects associated with the laser treatment including erythema, burning, pain, and itching were collected. Photographs were obtained using identical digital camera settings, lighting conditions, and patient positioning at every consultation.

Objective evaluations of each scar were collected before and after every treatment through the following instruments: [A] Scar thickness using a doppler ultrasound DP-6600 digital ultrasonic diagnostic imaging system (Mindray, Redmond, WA) with a 10 MHz frequency probe; [B] Scar firmness was assessed with a Cutometer dual MPA 580 (Courage Khazaka Electronic GmbH, Germany) mounted with a 6 mm handheld probe. The Cutometer uses a principle based on suction and elongation measurements, generating a negative pressure (set at 500 mbar for 1 s suction and relaxation times) which draws skin into the probe’s central aperture and estimates skin penetration depth using an optical measuring system^19^; [C] Scar pigmentation with a Mexameter MX 18 connected to the Multiprobe Adapter System (Courage Khazaka Electronic GmbH, Köln, Germany). The device measures pigmentation of the scar expressed by Melanin index^19^; [D] Blood supply of the scar were examined using a blood perfusion imager PeriCam PSI System (Perimed, Järfälla, Sweden) and Mexameter MX 18 (Courage Khazaka Electronic GmbH, Germany)^20^. Both were applied to detect changes of scar vascularity. The PeriCam PSI System is a blood perfusion imager 70-mW system based on Laser Speckle Contrast Analysis (LASCA) technology utilizing a laser wavelength of 785 nm^20^. The Mexameter MX 18 uses an erythema index to represent the quantity of haemoglobin at the detection site^19^. The Cutometer and Mexameter measurements were measured over three areas which were repeated three times. The first point was measured at the proximal 1/3 along the axis of the keloid or hypertrophic scars. The second point was measured in the middle of the scar and the final measurement was taken at the distal 1/3 of the scar. The mean average of the three detection points were then calculated to achieve an overall mean result.

### Laser technique

A fractional non-ablative laser LASEmaR 1500 (EUFOTON, Trieste, Italy) with a semiconductor gallium arsenide (GaAs) emitting 1470 nm wavelengths of light, was adopted in this study. The energy was delivered through a disposable optical bare fibre with a 300-micron diameter. The power output was set according to scar hardness in order to penetrate the scar with minimal resistance, generally starting from 3 W and gradually increasing to a maximum power output of 6 W if necessary, with a maximum fluence 999.9 kJ/cm^2^. The optical fibre was initiated with the laser to penetrate and deliver the energy through 1 to 4 passes of the scar. Entry points with a distance of 2–4 mm were made surrounding the scar (Video 1). The procedure was repeated once every one to two months until satisfactory results were achieved for the clinician and patient.

### Statistical analysis

All data were initially entered into an Excel database (Microsoft, Redmond, Washington—United States) and the analysis was performed using the Statistical Package for the Social Sciences Windows, version 15.0 (SPSS, Chicago, Illinois, USA).

The paired t-test was used to compare the changes in scar thickness, scar firmness, scar pigmentation, and scar blood supply before and after the laser treatment.

Subjective assessment before and after laser treatment were compared using Chi-square test.

Descriptive statistics consisted of the mean ± standard deviation (SD) for continuous parameters with normal distributions (after confirmation with histograms and the Kolmogorov–Smirnov test).

Comparison among groups was performed with the Student T-test for paired data or non-parametric Chi-Square test or Fisher’s exact test (if cells < 5) for categorical data. A *p* value < 0.05 was considered statistically significant. To evaluate the minimum sample size, a “power analysis” was conducted, which considered a downward difference of 25% from the average parameter of HS Thickness between pre and post treatment. Moreover, a level of 95% (alpha = 5%) for a 1-tailed test, a power of 82% was accepted with a sample size of 9 patients.

### Ethics

The study was conducted according to the criteria set by the declaration of Helsinki (1964) and successive modifications. The study protocol was approved by the local institutional ethics review committee (Jiao Tong University-Ninth People's Hospital, Shanghai). Clinical trial registration number: ChiCTR2000038092 (http://www.chictr.org/en/), last update 10/09/2020. Written informed consent was obtained from each patient. Informed consent from a parent and/or legal guardian was obtained for patients under the age of 18 to participate in this study.

## Results

### Patient characteristics

A total of twenty-one patients (mean age 28.4 years, range 3–66) with HSK were enrolled in this study which we divided in two subgroups. The first subgroup contained nine patients who had HS whilst the second subgroup contained twelve patients with keloids. Fourteen patients were male and seven females. Locations of the scars were chest (9), back (5), flank (4), thigh (4), shoulder (3), dorsal foot (2), arm (2), and other areas (6). The average scar size was 207.19cm^2^ (range 18.00–1063.50 cm^2^). The aetiology of scars was trauma (7 patients), burns (6 patients), acne scarring (4 patients), furuncle (3 patients), and previous surgery (1 patient). The average number of treatments was 1.38 (range 1–3). The mean single treatment energy was 4486.76 J (range 388–17536 J) and mean single treatment power was 4 W (range 3–6 W). Average follow-up was 7.2 months (range 6–12 months) (Table 1).

**Table 1:** Patient characteristics.

*n*	Sex	Age (years)	Fitz Patrick skin type	Scar location	Scar size (cm^2^)	Aetiology	Total treatment (session)	Energy for single treatment (J)	Power for single treatment (W)	Follow-up (month)	Type of scar
1	F	3	II	Elbow, flank, thigh and leg	1063.5	Burn scar	1	13,176	4	7	Hypertrophic scar
2	M	8	III	Flank and thigh	120.5	Trauma	1	2892	4	9	Keloid
3	M	9	III	Back	120	Burn scar	2	3058	4	6	Hypertrophic scar
4	M	11	IV	Anterior chest	45	Furuncle	2	2010	3	10	Keloid
5	F	20	III	Knee	36	Trauma	1	901	4	6	Hypertrophic scar
6	M	56	IV	Ear, back and shoulder	541.9	Burn scar	1	11,515	5	6	Keloid
7	F	18	III	Dorsal foot	28	Trauma	2	617	4	9	Keloid
8	M	46	IV	Arm and chest	261	Burn scar	3	3871	4	6	Keloid
9	M	5	III	Back, flank and thigh	284	Trauma	2	8623	5	8	Hypertrophic scar
10	M	40	IV	Anterior chest	180	Acne scar	1	7561	4	10	Keloid
11	M	35	III	Chest, back and arm	842	Furuncle	1	17,536	6	6	Keloid
12	F	48	III	Anterior chest	18	Furuncle	1	388	3	10	Keloid
13	F	7	IV	Shoulder and chest	36	Burn scar	1	828	3	7	Keloid
14	M	3	II	Flank, back and thigh	447	Burn scar	1	7687	3	6	Hypertrophic scar
15	M	17	V	Shoulder	45	Surgery	1	622	4	7	Hypertrophic scar
16	F	23	IV	Dorsal foot	24	Trauma	1	1097	4	6	Hypertrophic scar
17	M	47	IV	Anterior chest	70	Acne scar	1	3527	4	6	Keloid
18	M	55	III	Anterior chest	77	Acne scar	1	3169	4	6	Keloid
19	F	49	IV	Dorsal hand	26	Trauma	2	848	4	6	Hypertrophic scar
20	M	31	IV	Palm	20	Trauma	1	1308	4	8	Hypertrophic scar
21	M	66	IV	Anterior chest	66	Acne scar	2	2988	4	6	Keloid

### Objective assessment

The scar thickness was assessed at each visit and the doppler showed a decrease of 0.308 ± 0.138 cm (*p* < 0.05) at 6 months. Scar thickness significantly reduced compared to the baseline (0.942 ± 0.377 cm vs. 0.633 ± 0.306 cm). In particular, the two subgroups showed a 27.7% and 28.2% reduction in scar thickness of HS and Keloid scar.

The scar firmness was evaluated at each visit and the Cutometer tester (R0 and Q0 parameters of hardness are inversely proportional to its numerical size) measured a change respectively of − 0.023 ± 0.008 (*p* = 0.007) and − 2.616 ± 1.169 Units (*p* = 0.029). The scar firmness was improved (R0: 1.816 ± 0.055 Units, Q0: 365.629 ± 13.113 Units vs. R0:1.838 ± 0.057 Units, Q0:368.244 ± 12.442 Units). Respectively, the two subgroups showed a significant improvement of 1.2% (*p* < 0.05) in HS and an improvement of 0.4% (*p* = 0.26) in keloids which was not statistically significant.

Regarding the scar pigmentation parameters, from the two subgroups combined there was no significant improvement in scar pigmentation. The Melanin Index before the treatment was 251.413 ± 157.716 Units, and after 6 months was 234.349 ± 90.708 Units with a difference of 17.063 ± 131.33 Units (*p* = 0.308). However, the keloid group independently showed a 21.3% (*p* < 0.01) improvement in scar pigmentation.

The scar vascularity was measured by PeriCam PSI blood perfusion imager that indicated a decrease of blood perfusion volume in the scar of 33.645 ± 15.667 Units and by the Mexameter that showed the Erythema index decreasing 17.349 ± 8.959 Units. This indicates that the laser significantly reduces the blood supply of the scar (PeriCam PSI: 117.888 ± 44.593 vs. 84.243 ± 33.244;* p* < 0.05. Erythema Index: 437.714 ± 96.771vs. 420.365 ± 96.475; *p* < 0.05). Furthermore, the two subgroups independently showed a significant reduction of blood perfusion at 29.6% and erythema index at 4% in HS and a reduction of blood perfusion at 22.7% and erythema index at 3.1% in keloids.

The overall results are summarized in Table 2, whereas the independent findings of each subgroups are showed in Table 3.

**Table 2:** Scar modifications, pre and post laser treatment.

Features	Pre-operatively	Post-operatively	Pre-operatively/post-operatively	*p*
Scar thickness (cm)	0.942 ± 0.377	0.633 ± 0.306	0.308 ± 0.138	**< 0.001**
**Scar firmness**				
R0 (Units)	1.816 ± 0.055	1.838 ± 0.057	− 0.023 ± 0.008	***0.007***
Q0 (Units)	365.629 ± 13.113	368.244 ± 12.442	− 2.616 ± 1.169	***0.029***
**Scar pigmentation (units)**				
Melanin index	251.413 ± 157.716	234.349 ± 90.708	17.063 ± 131.33	*0.308*
**Scar blood supply**				
Blood perfusion (units)	117.888 ± 44.593	84.243 ± 33.244	33.645 ± 15.667	**< 0.001**
Erythema index (units)	437.714 ± 96.771	420.365 ± 96.475	17.349 ± 8.959	**< 0.001**
**VSS**^**a**^** scores**				
Pigmentation	2.476 ± 0.680	2.333 ± 0.658	0.143 ± 0.359	*0.083*
Height	4.000 ± 0.000	2.000 ± 0.617	2.000 ± 0.617	**< 0.001**
Vascularity	2.762 ± 0.436	1.381 ± 0.498	1.381 ± 0.498	**< 0.001**
Pliability	3.524 ± 0.512	2.000 ± 0.775	1.524 ± 0.602	**< 0.001**

^a^Vancouver scar scale.

**Table 3:** Hypertrophic scar and keloid subgroups: modifications and improvement percentages, pre and post laser treatment.

	Thickness		Thickness		Firmness		Firmness	
	HS		Keloid		HS		Keloid	
	Pre	Post	Pre	Post	Pre	Post	Pre	Post
N	9 (× 3 = 27)	9 (× 3 = 27)	12 (× 3 = 36)	12 (× 3 = 36)	9 (× 3 = 27)	9 (× 3 = 27)	12 (× 3 = 36)	12 (× 3 = 36)
Mean ± SD	0.83 ± 0.23	0.60 ± 0.20	1.03 ± 0.45	0.74 ± 0.45	368.7 ± 15.9	373 ± 16.7	363.3 ± 10.2	364.7 ± 5.9
Min; Max	0.45;0.33	0.40;1.10	0.45;2.00	0.20;1.84	345;421	353;426	344;380	352;374
Difference	− 0.23		− 0.29		4.3		1.4	
% improve	− **27**.**7**		− **28**.**2**		**1**.**2**		**0**.**4**	
T-test paired (*p*)	**< 0**.***001***		**< 0**.***001***		**< 0**.***05***		** = 0**.***26***	

	Blood perfusion		Blood perfusion		Erythema index		Erythema index	
	HS		keloid		HS		Keloid	
	Pre	Post	Pre	Post	Pre	Post	d Pre	Post
N	9	9	12	12	9 (× 3 = 27)	9 (× 3 = 27)	12 (× 3 = 36)	12 (× 3 = 36)
Mean ± SD	99.9 ± 29.6	70.3 ± 23	131.4 ± 50.1	101.6 ± 43.3	421.9 ± 108.1	404.9 ± 107.4	449.6 ± 87	435.8 ± 93.2
Min; Max	31;142	22;102	30;228	20;189	243;632	224;621	167;576	144;651
Difference	− 29.6		− 29.8		− 17.0		− 13.8	
% improve	− **29**.**6**		− **22**.**7**		− **4**.**0**		− **3**.**1**	
T-test paired (*p*)	**< 0**.***001***		**< 0**.***001***		**< 0**.***001***		**< 0**.***05***	

	Pigmentation		Pigmentation		VSS total score		VSS total score	
	HS		Keloid		HS		Keloid	
	Pre	Post	Pre	Post	Pre	Post	Pre	Post
N	9 (× 3 = 27)	9 (× 3 = 27)	12 (× 3 = 36)	12 (× 3 = 36)	9	9	12	12
Mean ± SD	201.7 ± 108.2	243.8 ± 101.1	288.7 ± 179	227.3 ± 82.9	12.4 ± 0.9	7.2 ± 1.6	13.0 ± 0.7	8.1 ± 2.1
Min; Max	82;486	82;471	82;626	82;327	11;14	5;10	12;14	5;11
Difference	42.1		− 61.4		− 5.2		− 4.9	
% improve	**20**.**9**		− **21**.**3**		− **42**.**0**		− **37**.**9**	
T-test paired (*p*)	**= 0**.***06***		**< 0**.***01***		**< 0**.***001***		**< 0**.***001***	

### Subjective assessment

Pigmentation, Height, Vascularity and Pliability were assessed according to the VSS score. The overall findings are reported in Table 2, whilst independent analysis of each subgroup is summarized in Table 3. Results showed that the laser therapy can significantly reduce the blood supply, decrease thickness and enhance pliability of HSK. In particular, there was an improvement in the total VSS score of 42% in the HS subgroup and 37.9% in the keloid subgroup.

**Figure 1:**
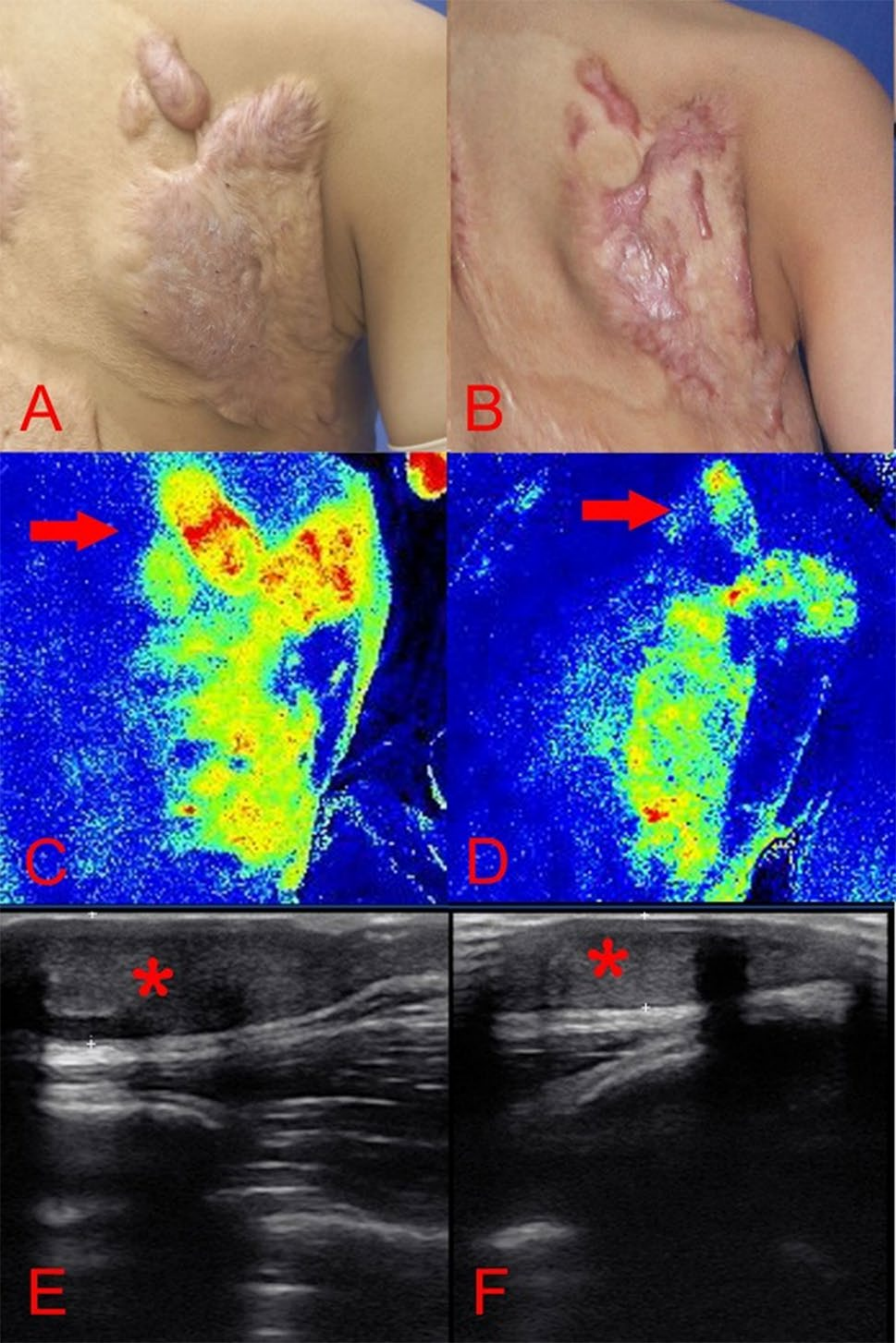
A 9-year-old boy [case 3] with a hypertrophic scar on his back prior laser treatment (**A**); 6 months post two laser therapy sessions (**B**); the scar blood perfusion showed 140.86 Units prior to treatment (**C**), a 32.64% reduction of the blood supply to the scar post laser treatment (**D**); the average thickness was 1.05 cm before the operation (**E**); a 63.81% decrease of scar thickness post laser treatment (**F**).

**Figure 2:**
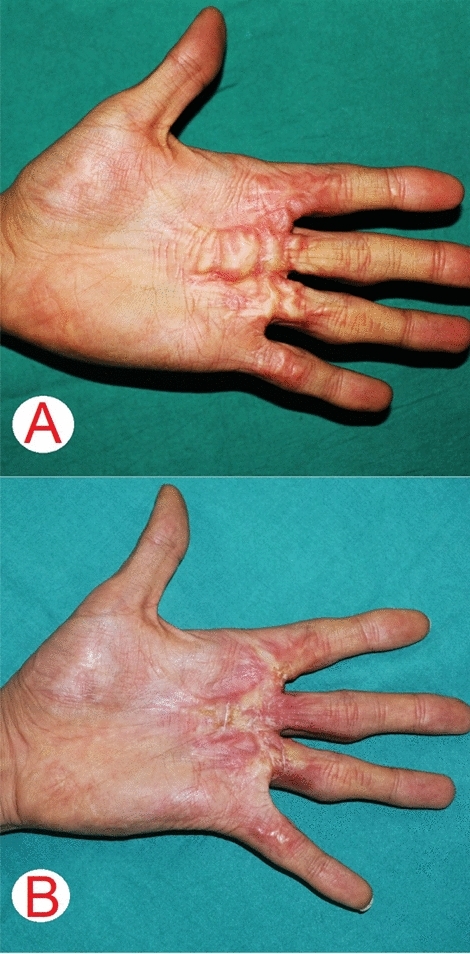
A 31-year-old man [case 20] had a hypertrophic scar on his left palm after sustaining a traumatic injury over 1.5-year ago (**A**); post one session of laser treatment (**B**).

**Figure 3:**
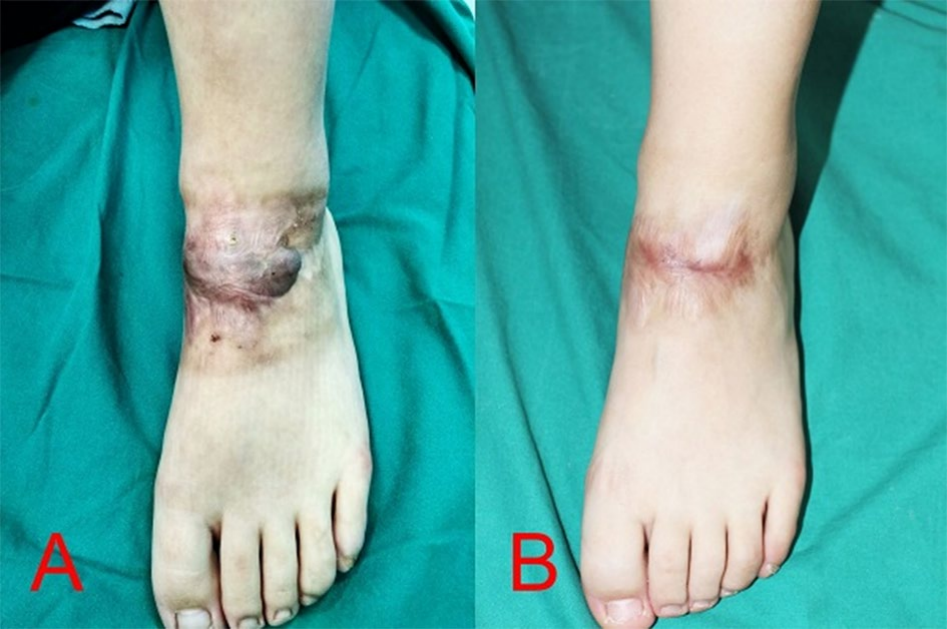
An 18-years-old girl [case 7] sustained a keloid scar post traumatic injury on the dorsum of her left foot for the duration of 1 year (**A**); post two sessions using intralesional 1470 nm bare-fibre diode laser (**B**).

**Figure 4:**
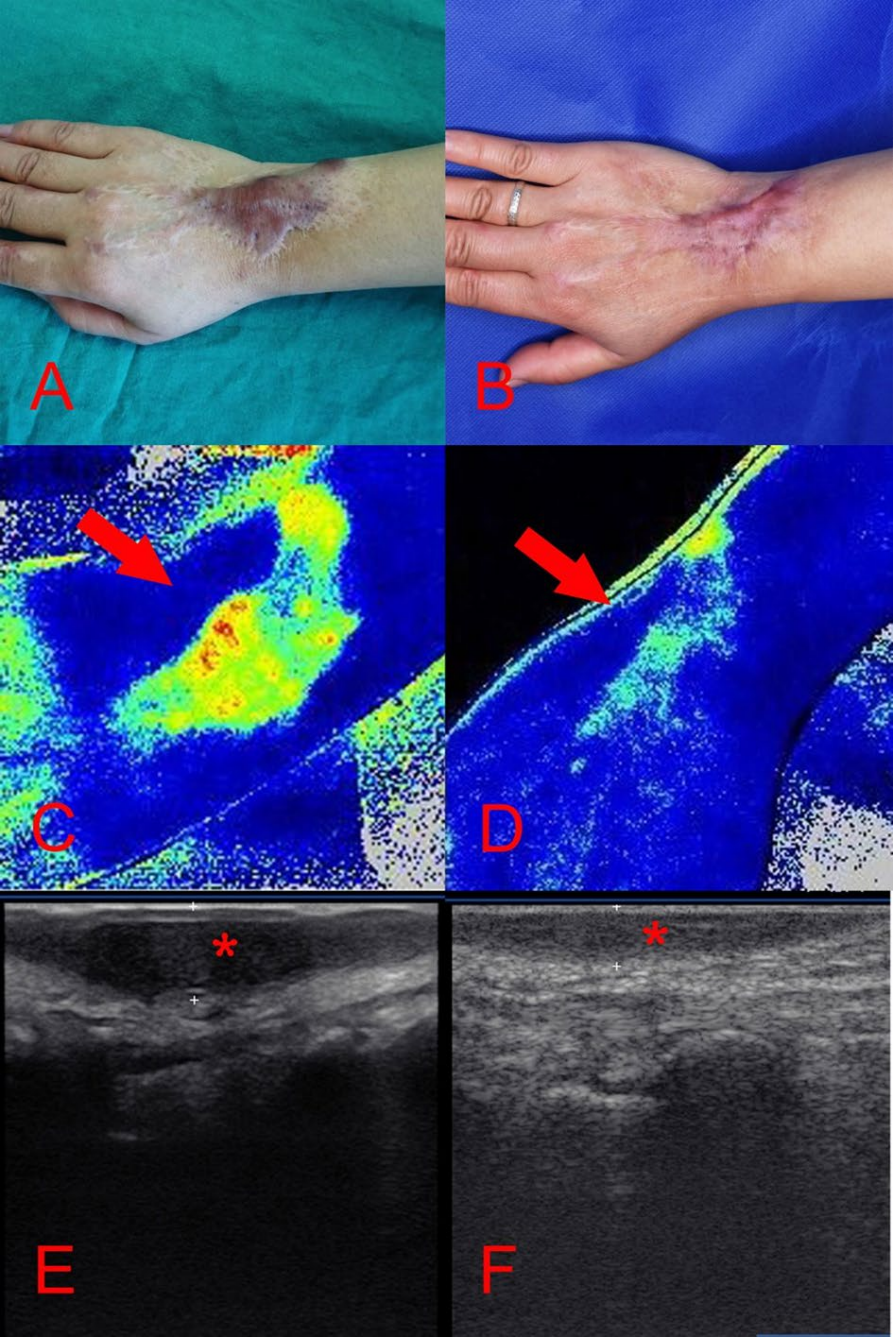
A 49-years-old woman [case 19] sustained a post-traumatic hypertrophic scar over 2-years prior to treatment (**A**); after 6 months follow-up and 2 laser sessions (**B**); the scar blood perfusion was significantly decreased: prior the operation was 104.29 Units (**C**), post operation there was a reduction of 28.46% (**D**); the scars thickness prior to the operation was 0.73 cm (**E**), post operation was decreased 43.84% (**F**).

**Figure 5:**
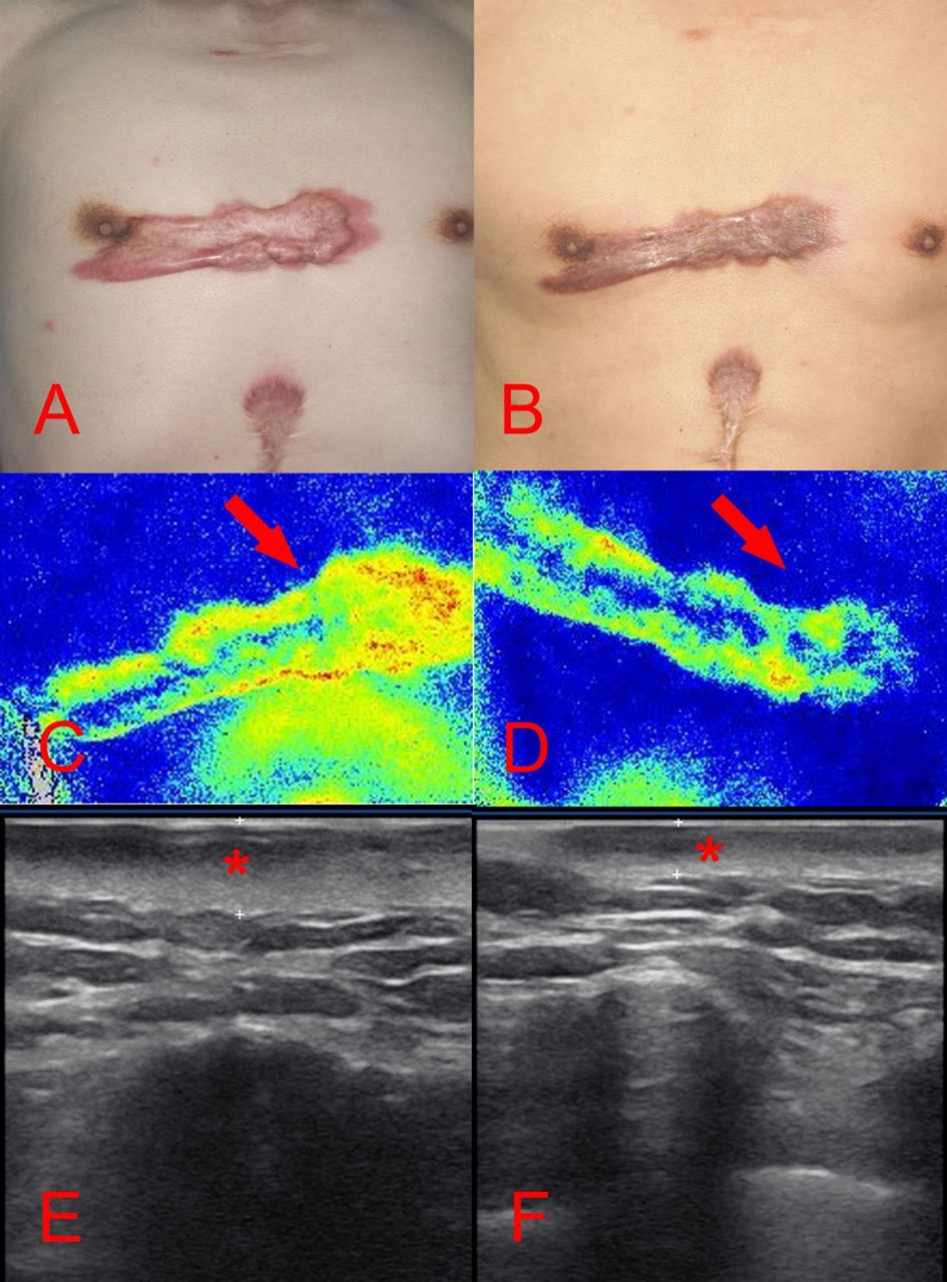
A 66-years-old man [case 21] sustained a keloid on his chest caused by acne over 10 years period (**A**); after a 6 months follow-up and 2 treatments using intralesional 1470 nm bare-fibre diode laser (**B**); the scar blood perfusion showed 98.79 Units before the treatment (**C**), which decreased to 38.92% after two sessions of laser treatment (**D**); the average thickness of the scar was 0.79 cm (**E**), resulting in a decreased of 45.57% after the laser treatment (**F**).

**Figure 6:**
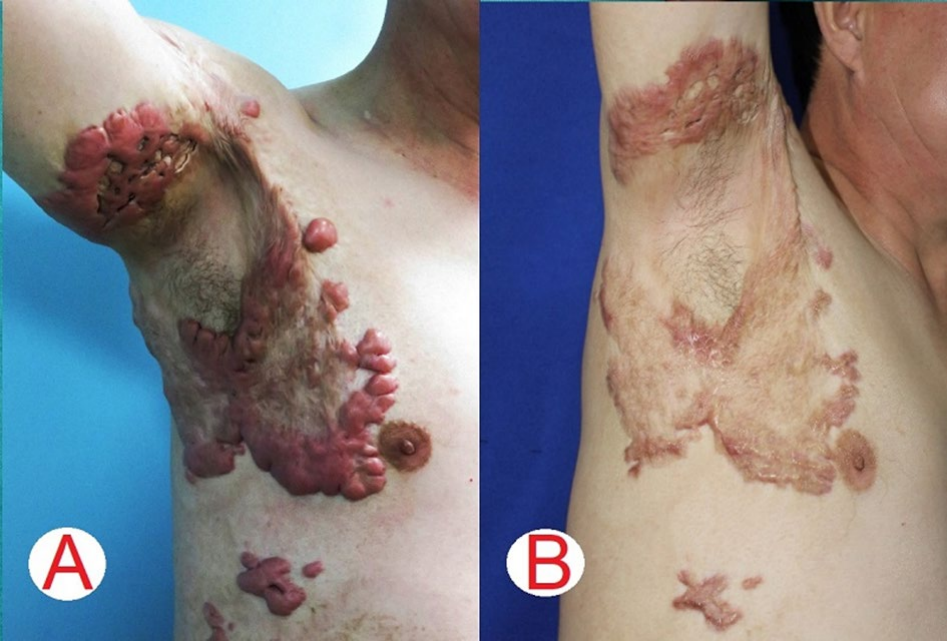
A 46-years-old man [case 8] had a keloid burn scar over a 2-year period, which extended to his chest and arm (**A**); after a 6 months follow-up and 3 laser sessions the patient achieved significant cosmetic and functional outcomes (**B**).

Photographs of representative patients are shown in Figs. 1, 2, 3, 4, 5 and 6.

Incidence of postoperative pain was 85.71%, duration was 2.19 ± 0.75 days, pain degree score was 4.19 ± 1.23Units, local swelling rate was 100% and the duration was 7.00 ± 1.10 days. The rate of partial scar tissue necrosis was 28.57%, healing time was 24.83 ± 12.05 day. None of the patients after laser procedure showed hypertrophy during the 6 to 12 months of follow-up period. None reported heavy pigmentation > 3 months, hypopigmentation or skin infection. This data is summarized in Table 4. The percentage of patients who were very satisfied or satisfied was over 85%.

**Table 4:** Adverse events after treatment using intralesional 1470 nm bare-fibre diode laser.

Adverse events	%/Mean ± SD	Duration
Pain	85.71%	2.19 ± 0.75 days
VAS scores for pain evaluation	4.19 ± 1.23 Units	–
Oedema	100%	7.00 ± 1.10 days
Partial scar necrosis	28.57%	24.83 ± 12.05 days
Hyperpigmentation > 3 months	0	0
Hypopigmentation	0	0
Skin infection	0	0
Exacerbation of keloid or hypertrophic scar	0	0

*SD* standard deviation, *VAS* visual analogue scale.

## Discussion

Several innovative strategies have been introduced for the treatment of HSK over the past few decades^11–16^. The evolution of laser treatment has shown a wide range of clinical and cosmetic applications including its use in the treatment of different types of scars.

In the 1980’s, Castro et al.^21^ reported first experiments analysing the effects of the Nd:YAG laser on human skin fibroblasts. Despite encouraging early reports, subsequent studies showed limited efficacy and a high incidence of side effects^22^. Other authors used the continuous wave CO2 laser (10,600 nm) to selectively inhibit collagen production^23^. However, initial results were poor and further studies showed that keloid formation was not inhibited after 1 year^22–24^. Thanks to the introduction of selective photothermolysis on the microvasculature of the scars, pulsed lasers were used to provide targeted selectivity with limited thermal injury^25^.

Recent advances in the use of fractionated lasers, based on the concept of using fractional photothermolysis showed faster epidermal tissue repair^26^. Thus, using columns of coagulation in microthermal zones has no destructive effect on the epidermis, leaving the skin intact between the small cores of coagulation, preserving the integrity of the epidermis^27^.

New tools were developed based on these innovative devices, to deliver the laser energy. A laser diode system LASEmaR 1500, at the wavelength of 1470 nm, was used as the laser energy source, near a peak in the water absorption spectrum. Laser energy was delivered through a specific type of optical bare fibre with 300-micron diameter. The fibre, with directional laser irradiation, enables penetration through tissue to a depth of 2–3 mm, enabling deeper penetration of the non-ablative fractional laser when treating thicker scars, an issue inherent with this type of laser in comparison to longer wavelength ablative fractional lasers^28^. The heat produced from this laser is emitted in a narrow field, so water quickly vaporizes in the surrounding tissue, resulting in selected tissue ablation and coagulation, with localised cell lysis and tissue necrosis. These kinds of fibre laser are widely adopted in vascular surgery for the treatment of endovenous ablation of incompetent saphenous veins and varicose veins^29,30^. The use of fibre laser has rapidly expanded in other disciplines such as urology, for the treatment of prostate hyperplasia^14,15^, otorhinolaryngology, for the treatment turbinate hypertrophy^31^, colorectal surgery for the treatment of anorectal fistula^32^, plastic surgery, for body contouring and lymphedema treatment^33,34^.

To our knowledge, we were the first in adopting this technique for scar therapy^13^, and have now evaluated its efficacy and safety in a larger cohort of patients for the treatment of HSK.

Our study showed that the 1470 nm laser can limit inflammation and decrease perfusion therefore suppressing the development of aberrant scars by effectively reducing the thickness (almost 30%) and inflammation of HSK (*p* < 0.05).

Different studies have demonstrated that the denaturation temperature of collagen is 60–70 °C, and the carbonization temperature is above 200 °C^35^. The temperature of the tissue surrounding the optical fibre tip measures between 100 and 200 °C, allowing the breakdown of collagen deposits and the extracellular matrix. The collagen of cicatricial tissue degenerates and shrinks whilst diffused heat leads to protein denaturation and tissue coagulation^36^. Albergel et al.^37^, demonstrated that HSK produce more collagen and an extracellular matrix, increasing scar tissue density and collagen type I and III, forming fibrotic cords that increase firmness and decrease elasticity. Furthermore, Miles et al.^38^ analysed the collagen composition highlighting the triple helical structure that is stabilized by 4-hydroxyproline which forms a hydrogen bond. This triple helical structure is strongly associated with the stability of the collagen. When the collagen is heated, the triple helix structure changes into amorphous random coils, which causes the collagen contraction by up to one-third and modifies the collagen tensile strength and viscoelasticity^39^.

Our study showed that the scar firmness index was reduced after fibre laser treatment, leading to softer tissue, therefore improving elasticity of the scar (*p* < 0.05). The HS subgroup showed a significant improvement of 1.2% (*p* < 0.05) while the keloid subgroup an improvement of 0.4% (*p* = 0.26) was not statistically significant. This suggests a modification of the scar tissue texture. Thermal effects change cross-linkage between scar collagen and modify the three helical collagen structures, leading to collagen contraction and degeneration.

Post-operative hyperpigmentation, discoloration or prolonged erythema are the most common adverse effect after scar laser therapy^22,27,40^. Our study found that the 1470 nm fibre laser therapy does not intensify the melanin deposition in the keloids scar, therefore cannot create hyperpigmentation. In particular keloids had a significant reduction of pigmentation at 21.3%. This indicates that melanin is not absorbed by the 1470 nm wavelength. Moreover, the fibre laser may destroy melanin deposits, reducing the pigmentation of the scar.

Different authors reported that the proliferation of fibroblasts, collagen synthesis, cell activities and metabolism is enhanced in HSK, stimulating new capillaries that transport nutrients. Ogawa, et al.^11,41^ noted that angiogenic cytokine VEGF levels and vascular density in HSK were significantly higher than normal tissue. This assumes that the blood supply is the most important factor for the nutrients of the scar tissue. Investigating changes on scar vascular supply through erythema index and PeriCam PSI, we report that the scar blood perfusion was significantly decreased (*p* < 0.001). The erythema of the scar, as assessed by colorimetry, showed a significant difference in comparison to the preoperative status. In particular, blood perfusion had a reduction of 29.6% in HS and 22.7% in keloids; the erythema index also has an improvement of 4% in HS and 3.1% in keloids. This is in contrast to the controlled study of Wittenberg et al.^42^, where HSK treated by pulsed dye laser tended to lose the redness over a period of 1-year post operation. A possible explanation is that the fibre laser reduces the inflammatory period of the scar hence accelerates its maturation phase.

In this study we solely utilized a 1470 nm fibre laser to treat HSK and we achieved satisfactory results in terms of scar volume reduction, improving scar elasticity, decreasing erythema, pigmentation and scar vascularity. The penetration depth of the 1470 nm laser can only reach up to 2–3 mm. Thus, to treat scars that protrude the skin more than 2 mm multiple passes and treatments are necessary. Consequently, this laser method may be combined with other lasers or agents^28,43–46^.

There are a few limitations to this study which include a small sample size and the lack of a control group. Caution is warranted when interpreting subjective results as improvements can be seen as highly variable due to research observer bias. The scar measurements method can also be a limitation of the study. This is due to a reduction in diameter of the scar that could lead to the exact spot not being remeasured each time, leading to variability in the results. The therapy was shown to be safe with no adverse reactions or recurrence, although moderate pain was experienced for an average of two days by most patients. Finally, follow-up numbers and different timing of interventions between patients may have also had an impact on the reported results.

Future studies should address the optimal timing of treatment, the optimal laser parameters and settings and the combination of complimentary therapies to achieve higher satisfactory and long-term results^28^.

## Conclusion

The present study shows that the 1470 nm fibre laser could be a promising treatment modality for HSK. We have demonstrated that it is a safe and effective method, based on both subjective and objective analyses. It is an effective minimally invasive scar reduction therapy that may aid as an additional powerful tool in the surgeon's armamentarium. However, to confirm these results, additional investigations on the fibre laser mechanisms including immuno-histopathological studies are necessary.

## Supplementary information

Supplementary video.

Supplementary Information.

## Data Availability

The datasets generated during the current study are available from the corresponding author on reasonable request.
