# Supplementary material for: Treatment of hypertrophic scars and keloids using an intralesional 1470 nm bare-fibre diode laser: a novel efficient minimally-invasive technique
Source: Sci Rep. 2020 Dec 10;10:21694. doi: 10.1038/s41598-020-78738-9 (PMC7728807; doi:10.1038/s41598-020-78738-9)
Supplement: Supplementary file 2 — Supplementary Information. [file 41598_2020_78738_MOESM2_ESM.docx]

Video 1. The process using the intralesional 1470 nm bare-fibre diode laser for treating hypertrophic and keloid scars indicated the distance between two entry sites of 2 to 4mm and penetration of the fibre through the scar tissue.
